# Predictive modelling of brain disorders with magnetic resonance imaging: A systematic review of modelling practices, transparency, and interpretability in the use of convolutional neural networks

**DOI:** 10.1002/hbm.26521

**Published:** 2023-11-01

**Authors:** Shane O'Connell, Dara M. Cannon, Pilib Ó. Broin

**Affiliations:** ^1^ School of Mathematical and Statistical Sciences, College of Science and Engineering University of Galway Galway Ireland; ^2^ Clinical Neuroimaging Laboratory, Galway Neuroscience Centre, College of MedicineNursing and Health Sciences University of Galway Galway Ireland

**Keywords:** Alzheimer's disease, convolutional neural networks, deep learning, MRI

## Abstract

Brain disorders comprise several psychiatric and neurological disorders which can be characterized by impaired cognition, mood alteration, psychosis, depressive episodes, and neurodegeneration. Clinical diagnoses primarily rely on a combination of life history information and questionnaires, with a distinct lack of discriminative biomarkers in use for psychiatric disorders. Symptoms across brain conditions are associated with functional alterations of cognitive and emotional processes, which can correlate with anatomical variation; structural magnetic resonance imaging (MRI) data of the brain are therefore an important focus of research, particularly for predictive modelling. With the advent of large MRI data consortia (such as the Alzheimer's Disease Neuroimaging Initiative) facilitating a greater number of MRI‐based classification studies, convolutional neural networks (CNNs)—deep learning models well suited to image processing tasks—have become increasingly popular for research into brain conditions. This has resulted in a myriad of studies reporting impressive predictive performances, demonstrating the potential clinical value of deep learning systems. However, methodologies can vary widely across studies, making them difficult to compare and/or reproduce, potentially limiting their clinical application. Here, we conduct a qualitative systematic literature review of 55 studies carrying out CNN‐based predictive modelling of brain disorders using MRI data and evaluate them based on three principles—modelling practices, transparency, and interpretability. We propose several recommendations to enhance the potential for the integration of CNNs into clinical care.

## Graphical abstract

Predictive modelling of brain disorders using convolutional neural networks applied to structural neuroimaging data has become popular in recent years. We systematically review 55 papers in the field and evaluate their modelling practices, transparency, and considerations of interpretability.

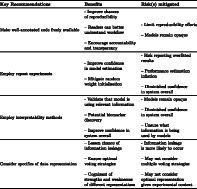

## 1 INTRODUCTION

Brain disorders, which include bipolar disorder, Alzheimer's disease, and schizophrenia, are a collection of debilitating neurological and psychiatric conditions characterized by a variety of features, including impaired cognition, altered mood states, psychosis, neurodegeneration, and memory loss (American Psychiatric Association, 2013). These phenotypes incur public and personal health burdens through reduced quality of life, social stigma, and increased mortality (American Psychiatric Association, 2013; James et al., 2018) and are therefore the focus of intense research. In particular, there is significant interest in building predictive models designed to differentiate conditions and their subtypes. Biomarkers identified using predictive modelling approaches could yield mechanistic insights into these diseases (Kupfer et al., 2008; Taber et al., 2010) offering the potential for early intervention and improved disease management (Shah & Scott, 2016). Magnetic resonance imaging (MRI) provides non‐invasive measures of brain structure and the increasing availability of large‐scale collections of MRI data has enabled a wealth of predictive modelling studies (Grover et al., 2015; Milham et al., 2017).

Previously, machine learning and classical statistical approaches have been used to highlight differential neuroanatomical patterns across several conditions, including subcortical structure volume reduction in bipolar disorder and Alzheimer's disease (Hibar et al., 2016; Roh et al., 2011). However, incorporating such information into clinical systems is non‐trivial, as the dynamics and limitations of a particular biomarker must be addressed prior to use (Carroll, 2013; Furiea & Gisele, 2009). Additionally, the methods used to identify discriminative features have their own considerations, such as requiring preprocessing tools to derive tabular brain summary information (Jenkinson, 2012; Reuter et al., 2012). These tools can produce variable results depending on the parameters chosen, even when applied to the same dataset, highlighting the importance of domain expertise in justifying data processing decisions (Botvinik‐Nezer et al., 2020). Additionally, statistical modelling often requires formal specification of expected variable relationships and is generally unsuited to high‐dimensional imaging data structures. Traditional machine learning approaches are also limited by their inability to consider spatial dependencies between groups of pixels, making it necessary to use tabular summary data. Deep learning algorithms have therefore become a popular methodology given their ability to consider arbitrarily complex relationships, providing greater model flexibility, and their lack of requirement for specification of expected variable relationships. Convolutional neural networks (CNNs) are deep learning models designed to detect spatial patterns in imaging data and have shown impressive predictive performance in various classification tasks. They have also been widely applied in the field of medical imaging for segmentation and prediction, particularly in the context of ageing and psychiatric/neurological disorder diagnosis (Kamnitsas et al., 2017; Simonyan & Zisserman, 2015; Ueda et al., 2019; Zou et al., 2017).

These recent developments have been enabled by access to large standardized neuroimaging data collections, such as the Alzheimer's Disease Neuroimaging Initiative (ADNI) (Jack Jr et al., 2008) and the UK Biobank (Littlejohns et al., 2020). However, there are a few caveats that bear consideration; firstly, deep learning models can suffer from multiple limitations, such as high parameter dimensionality, lack of interpretability, random weight initialization, lack of uncertainty, and difficulty to train (LeCun et al., 1995; LeCun et al., 2012; Yam & Chow, 2000; Zhang et al., 2018). Secondly, clinical decision systems require rigorous validation and reporting frameworks for more interpretable models; the use of opaque deep learning algorithms makes validation and transparency more difficult to achieve (Collins et al., 2015; Haibe‐Kains et al., 2020). Clinical decision systems that offer no explanation of an output are less likely to be incorporated into patient care frameworks. These factors combine to make the application of deep learning to clinical settings challenging.

As the number of studies applying deep learning to brain disorder prediction using neuroimaging data increases, the opportunity arises to examine factors which may limit their potential for clinical application. In this work, we systematically review 55 papers which report on such approaches. While many of the studies examined have been designed to demonstrate predictive capability, we sought to assess the existing literature with the aim of identifying key principles that can maximize the potential clinical value of future work. These principles are: (1) modelling practices, (2) transparency, and (3) interpretability. Below, we first provide a brief overview of CNNs and their workflow in the context of brain disorder imaging‐based models; we subsequently detail our motivation behind focusing on these three principles. We then analyze the selected articles in the context of these principles and propose several recommendations for future studies based on our results.

### 1.1 Convolutional neural networks

CNNs are a popular deep learning algorithm for many areas of biomedical imaging research, particularly those utilising MRI data (Hosseini‐Asl et al., 2018; Kamnitsas et al., 2017; Simonyan & Zisserman, 2015; Zou et al., 2017). Their structure is designed to account for spatial data patterns; this is accomplished through the use of filters and feature maps. A feature map is derived via *convolutional operations*, which are a matrix multiplication between a weight vector of arbitrary window size and an input image patch of the same size. Every number in the input window is multiplied by every number in the filter and summed together, providing the pixel value of a new feature map at the next layer. The convolution of the same filter over every patch of the input image generates the entire output feature map, which is usually the same size as the input image. Multiple feature maps are used in CNN architectures, each with its own filters, which, throughout model training, can detect distinct data patterns such as shapes and/or edges. CNNs build increasingly abstract representations of input data through iterative transformation operations with all variables at successive layers being the sum‐weighted combination of all previous layer outputs. Terminal fully connected layers provide a predictive output. Weight initialization is often random and training is carried out via backpropagation. A more in‐depth consideration of neural networks and their training can be found in LeCun et al. (1995) and (2012).

### 1.2 CNN implementations

**FIGURE 1:**
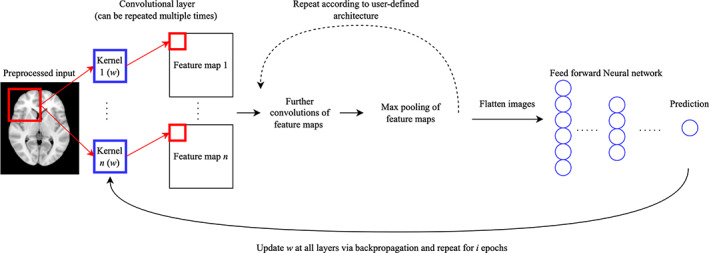
General experimental workflow. The preprocessed input image, either in 2‐ or 3‐dimensional format is passed to a CNN model (or ensemble of CNN models) for training and prediction, The weight vector, w, is updated via backpropagation at each epoch (training iteration), minimizing the error of the loss function chosen to train the model.

MRI‐based predictive modelling of neuroanatomical phenotypes with deep learning models generally follows the pipeline presented in Figure 1 or a variant thereof. Preprocessing is usually applied to skull strip, register raw input images, crop, resize, and/or contrast normalize. The preprocessed inputs are then used as training data for a CNN (or an ensemble of CNNs). Owing to the fact that many existing CNN models have been applied to 2D data domains, studies in the medical imaging field can adapt their data to fit existing architectures and make use of existing weights via transfer learning. Alternatively, researchers can train new models that operate on 3D data, as structural MRI scans are natively 3D (Billones et al., 2016; Weiss et al., 2016). Some studies also train custom architectures on 2D data (Aderghal et al., 2017; Barbaroux et al., 2020; Pelka et al., 2020). The CNN output is usually presented as a probability, which is then used to calculate performance metrics, such as the area under the receiver operating characteristic curve (AUC) and accuracy.

In the following sections, we define and justify our emphasis on modelling practices, transparency, and interpretability in the context of brain disorder classification using neuroimaging data. We note that these principles are domain‐agnostic and overlap with recent recommendations for improving the translational potential of machine learning and deep‐learning models (Walsh et al., 2021).

### 1.3 Modelling practices

Modelling practices here refers to the reliability of the methodology used. Studies that can be reproduced and that have attempted to mitigate factors that can influence the reliability of results are more likely to be integrated into clinical care settings. We examine the use of repeat experiments, the data splitting procedure, the reported accuracy, and the data representation strategy to evaluate this principle.

Repeat experiments ensure that the reported performance metrics are trustworthy across multiple random weight initializations and that the system as a whole can be expected to perform well if retrained. This is pertinent given that CNNs are parameter‐dense, making them more prone to overfitting. A useful type of repeat experiment includes *k*‐fold cross‐validation, whereby data is split into *k* folds and *k* − 1 folds are used to train the model with the *k*‐th fold serving as the testing set. This procedure is repeated *k* times until every fold has served as the testing set, providing an estimate of model performance across multiple data splits.

The reported accuracy is the final performance of the model as estimated from an evaluation strategy, which can include *k*‐fold cross‐validation, performance estimation on a separate test split within the same population, or estimation on a separate population. The overall capacity of a model to classify a brain disorder with fidelity in a generalizable manner will ultimately dictate its potential for clinical use.

The data representation strategy is of specific importance for CNN models in this domain, as structural MRI data is natively 3D, whereby each number is represented by a pixel. Thus, modelling entire volumes can be computationally expensive, and some studies may opt to split data into individual 2D slices. This comes with a set of considerations: firstly, each 2D slice is treated as an individual instance during conventional training procedures, meaning that performance metrics can either be reported per slice or combined to derive patient‐level quantities, prompting consideration of voting strategies; secondly, 2D data are more prone to information leakage if train‐test splitting is carried out after 2D slice derivation because the model may have been exposed to data from the same patient during training and evaluation. (information leakage), which can inflate performance estimates Additionally, studies may take multiple 3D patches per patient which may result in similar issues (Goldacre et al., 2019).

### 1.4 Transparency

Transparency refers to how clearly the study's methods are reported, including the use of code and model sharing. Several important advantages to code sharing have been described previously, including the facilitation of greater understanding of experimental outcomes and improved levels of reproducibility (Eglen et al., 2017; Markowetz, 2015). There are many hyperparameters associated with deep learning models, the choice of which can greatly impact predictive performance; clear and detailed reporting of these values and the mechanism of their choice is therefore an important aspect of model transparency as are comprehensive descriptions of model architecture and training schedules. Where possible, direct sharing of model weights is encouraged as it not only improves transparency but can also mitigate the large computational overhead of model training. In addition to model transparency, an explicit description of the data sources and demographics which yielded the reported results is vital not only to the reproducibility of a study but also to understanding any potential biases contained in the data.

### 1.5 Interpretability

Interpretability refers to the efforts made to explain features driving model predictions. Deep learning systems can be difficult to interpret, but efforts can be made to highlight image regions that are used during prediction to determine whether or not that information is relevant. This is particularly important as CNNs are prone to overfitting and can make use of any image feature, in turn making algorithmic biases likely (Hooker, 2021; Lepri et al., 2018). Ensuring that CNNs are using relevant information can increase confidence in the system. Models can be interpreted by saliency methods such as gradient‐based class activation mapping (Selvaraju et al., 2016; Simonyan & Zisserman, 2015). These approaches rely on deriving the gradient of a model's output with respect to input and weighting that quantity by the input—the final metric is then overlaid on the input for visualisation. This can indicate which regions are most ‘important' for prediction, but they are not directly comparable to coefficients from classical regression models. Another approach to understanding model decisions is the use of counterfactuals, which involves measuring changes in the predictive performance of a model when it is exposed to inputs with known qualities. An example of this would be noting the change in model output when a patient image with a thicker amygdala is used as the input (Keane & Smyth, 2020). We assess the interpretability of a study based on the use of methods that produce a saliency map (such as [Selvaraju et al., 2016] or [Simonyan & Zisserman, 2015]), which are gradient‐based, or which provide visualisation of internal feature map outputs.

## 2 METHODS

We conducted a systematic literature review according to PRISMA guidelines (Page et al., 2021), the details of which are provided below.

### 2.1 Inclusion/exclusion criteria

We limited our search to consider studies making use of traditional CNN architectures exclusively, whereby convolutional layer outputs, or other model outputs, are not used to train separate machine learning models. As these are the most common types of architecture, this better enables comparisons across studies. We also focused our attention on studies that use structural MRI data, as functional MRI data structures can often have different modelling requirements, including the use of time series methodologies that make them more difficult to compare between studies.

### 2.2 Search details

We performed a Web of Science (all databases) and Pubmed search with the following keywords.

((((structural) or (T1‐weighted)) AND (imaging)) AND ((MRI) OR (T1 MRI)) AND ((CNN) OR (convolutional neural network) OR (3D‐CNN))) AND (psychiatric OR depression OR autism OR bipolar OR Alzheimer's OR neurological) NOT (segmentation)

**FIGURE 2:**
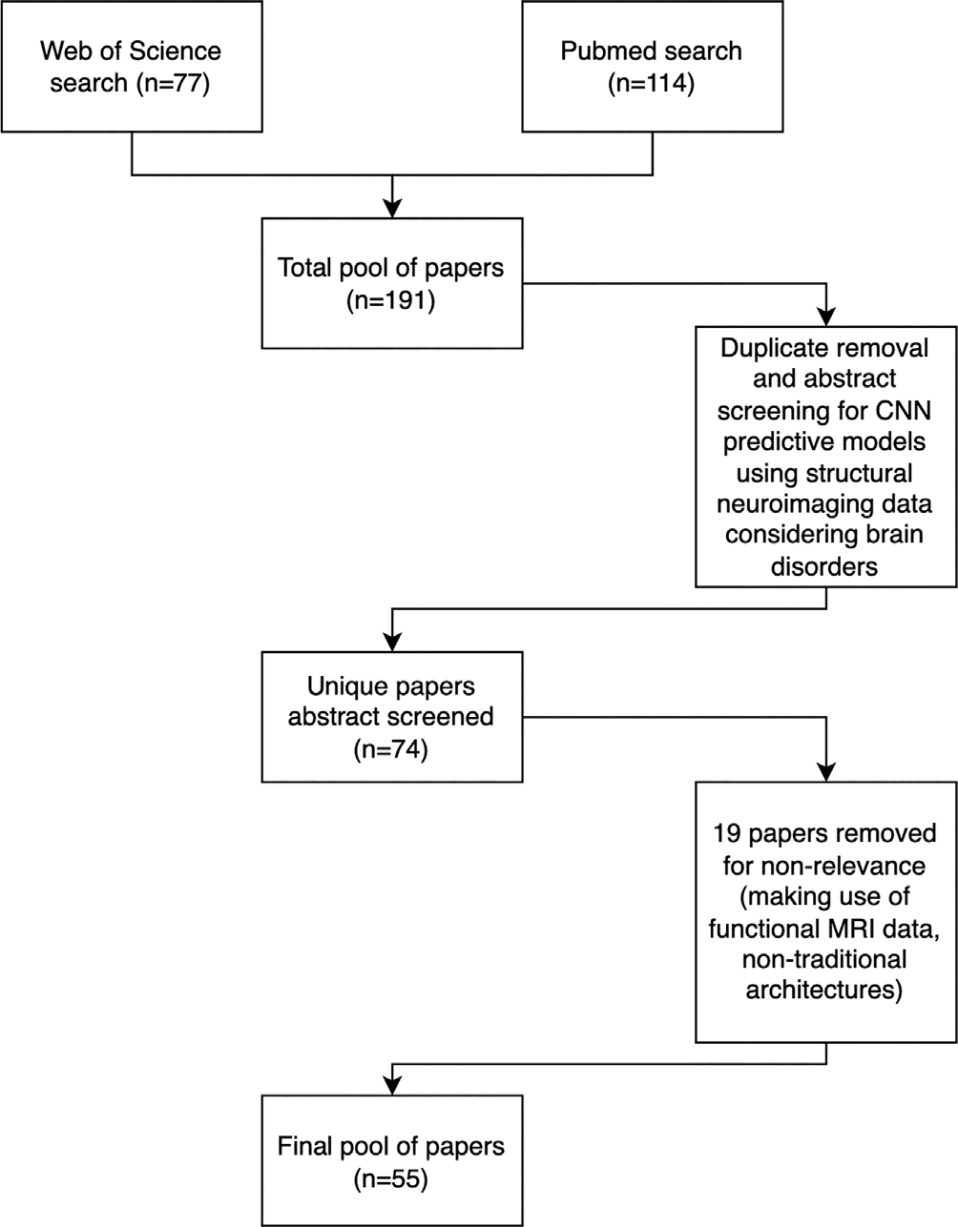
Flowchart detailing the article selection process.

For Web of Science, 77 results were returned, and for Pubmed 114 results were returned. Titles and abstracts were screened for relevance to the research question, and duplicates across both databases were removed, leaving a total of 74 papers. Nineteen studies were excluded for using functional MRI data and applying hybrid models where CNNs were not the primary modelling method; this resulted in a total of 55 papers remaining for review. The flowchart of this process is presented in Figure 2.

### 2.3 Desired variables

A standardized questionnaire was designed to evaluate the methodological details of the studies considered, including the presence or absence of repeat experiments, the overall data representation strategy, the reported accuracy, the sample size, the data source, whether or not an interpretability method was applied, and whether or not code was made available. To obtain accuracy measures, we recorded the highest performance in testing experiments as reported by authors for the main classification task. For example, certain studies made use of 3‐way classifiers for Alzheimer's disease, cognitive impairment, and controls—in these cases, we took only the Alzheimer's versus control reported accuracy. We marked sample size as NA where patient‐level data numbers were not reported. We used t‐tests to determine whether or not accuracy statistically varied across binary categories and carried out a linear regression to examine the relationship between accuracy and sample size. We also carried out a linear regression of accuracy against all measured variables (except code availability) to query their relationships with reported accuracy.

## 3 RESULTS

We organise our findings according to our three principles: modelling practices, transparency, and interpretability. The selected articles and their attributes can be found in Table 1, and a numerical summary of all results can be found in Table 2.

**TABLE 2:** Numeric summary of study attributes from the 55 papers satisfying selection criteria.

Question	Answer
How are data represented?	2D (*n* = 24), 3D (*n* = 31)
Is code available?	No (*n* = 49), Yes (*n* = 6)
Mean Accuracy (*μ* ± *STD*)	89.36 ± 8.694%
Is interpretability considered?	No (*n* = 38), Yes (*n* = 17)
Are there repeat experiments?	No (*n* = 25), Yes (*n* = 30)

### 3.1 Modelling practices across studies

We found that 24 out of 55 papers represented data in 2D format (Table 2). While this is more computationally efficient than 3D, it can make information leakage more likely. Accuracy calculation can be carried out per slice or per patient, introducing issues surrounding optimal voting strategies. Of the 24 studies making use of 2D slices, only one referred to voting methods (Ahmed et al., 2020). Several studies made use of single slices per patient (Aderghal et al., 2017; Herzog & Magoulas, 2021; Mendoza‐Léon et al., 2020). One paper making use of 2D slices provided code, detailing how individual 3D patient volumes were split into collections of 2D images (Sarraf et al., 2019).

**FIGURE 3:**
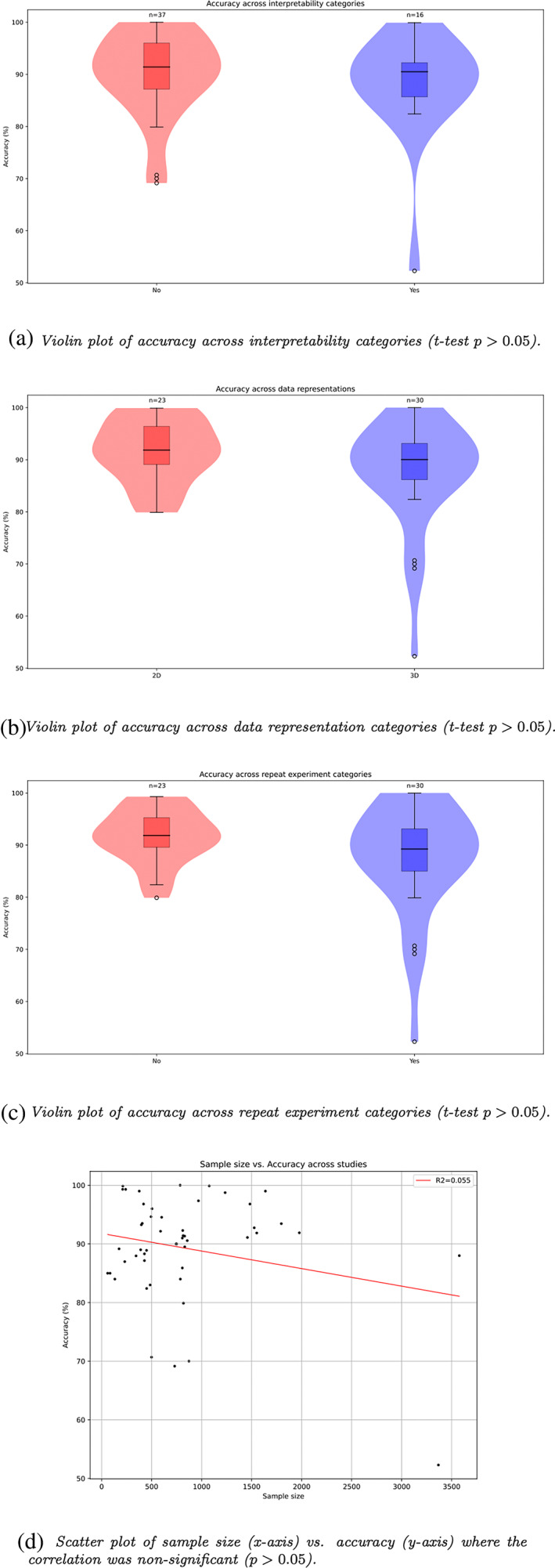
Plots of accuracy variation across binary categories (a = interpretability, b = representation, c = repeat experiments) and sample size (d). In plots a–c, studies where accuracy was not reported were excluded. (a) Violin plot of accuracy across interpretability categories (*t*‐test *p* > 0.05). (b) Violin plot of accuracy across data representation categories (*t*‐test *p* > 0.05). (c) Violin plot of accuracy across repeat experiment categories (*t*‐test *p* > 0.05). (d) Scatter plot of sample size (x‐axis) versus accuracy (y‐axis) where the correlation was non‐significant (*p* > 0.05).

We noted that 24 out of 55 studies made use of multiple models for training and prediction, with some papers using the output of one trained CNN as the input to another (Cui & Liu, 2019a; Li & Liu, 2018; Lian, Liu, Pan, & Shen, 2020; Liu, Zhang, Adeli, & Shen, 2018; Liu, Zhang, Nie, et al., 2018). This may impact generalization by increasing the chances of overfitting. Several studies used statistical tests to pre‐select informative image patches which can introduce bias by focusing the model on regions which may not be informative in full models (Liu, Zhang, Adeli, & Shen, 2018; Liu, Zhang, Nie, et al., 2018; Mendoza‐Léon et al., 2020). Furthermore, pre‐selecting regions based on accuracy metrics in one population may influence generalization capacity in another. In several studies, one model was trained on the whole dataset and the weights from that model were then used for transfer learning of another model with the same dataset, leading to potential leakage or overfitting (Ahmed et al., 2020; Folego et al., 2020; Lin et al., 2018; Mendoza‐Léon et al., 2020; Pelka et al., 2020). We note that while overfitting mitigation strategies can be employed, in cases where the weight training has been informed by access to testing labels, no degree of post‐leakage mitigation can remedy these specific effects. Thirty out of 55 studies employed repeat experiments, 10 of which reported only point estimate performance metrics. We found that the mean accuracy across all studies was 89.36 ± 8.694% (*μ* ± *STD*) and that there was no significant difference between reported accuracy metrics in any questionnaire results based on t‐tests and linear regression, however, accuracy did appear to be inversely correlated with increased sample size (Figure 3). Further, a regression of accuracy against all variables yielded non‐significant test statistics for every coefficient. The mean sample size of the 55 studies was ≈828 ± 691.

### 3.2 Transparency and interpretability considerations across studies

We found that 49 out of 55 papers did not provide code or model weights, meaning that the majority of studies relied on textual methods summaries. This implies limited methodological transparency which is an issue considering how modelling choices can impact system performance. Studies providing code facilitate clear, reproducible experimental practices (Böhle et al., 2019; Folego et al., 2020; Hu et al., 2021; Qiu et al., 2020; Sarraf et al., 2019; Spasov et al., 2019). Forty‐four of the 55 studies made use of the ADNI dataset during either training or testing. Every study made explicit mention of the database from which their samples came and textually described their preprocessing pipelin.

Seventeen of 55 studies considered interpretability by applying a saliency method (Selvaraju et al., 2016) or visualising feature maps (Simonyan & Zisserman, 2015). Of these 17, four articles discussed their interpretation of interpretability outputs in their findings (Böhle et al., 2019; Liu, Cheng, Wang, et al., 2018; Nigri et al., 2020; Qiu et al., 2020). Five of the 17 articles employing interpretability methods provided code, however only three code releases contained information on saliency method implementation (Böhle et al., 2019; Hu et al., 2021; Qiu et al., 2020).

## 4 DISCUSSION

Below, we discuss the findings summarized in Table 2 and propose several recommendations to maximize the potential clinical value of future studies making use of CNNs to predict brain disorders from structural neuroimaging data.

### 4.1 Data representation

A majority of papers made use of 3D data representations, either by deriving multiple patches per patient of arbitrary sizes or modelling entire brain volumes (patch‐based/region‐of‐interest‐based). Using entire brain volumes on a subject level can reduce the chances of information leakage while preserving one volume per patient, but is computationally expensive. Modelling multiple 3D patches per patient can potentially lead to information leakage if patch derivation is carried out before patient‐level train‐test splitting, and testing accuracies can be represented by different voting strategies. While 3D patch modelling can consider the spatial dependencies across 3 axes of brain data, it is unclear as to the benefits of focusing on smaller 3D regions compared to entire 2D images along one axis. A significant minority of papers made use of 2D data structures (24/57), which provide an attractive alternative considering the high computational burden of modelling in 3D and the ability to capture all brain information along one dimension. Workflows making use of multiple 2D images per patient can however be prone to information leakage and may represent testing accuracies by different means, thus requiring a greater level of care. We found that there were no statistically significant differences between reported accuracies across data representations (2D = 91.5 ± 6%, 3D = 88 ± 9%). This suggests that if performance inflation has occurred, it does not appear to be enriched for a specific data representation strategy. Nevertheless, researchers should be cognizant of the individual limitations associated with each experimental approach and proactively address issues where possible. For example, information leakage can be mitigated by ensuring slice (or patch) conversion post‐data splitting at the patient level, which can be verified by providing well‐annotated code. Additionally, where multiple slices or patches have been used per patient, the voting strategy should be explicitly detailed.

Utilizing single 2D slices may lead to performance estimation inflation because there is no guarantee the same biological information is being considered per patient at the same slice index. Several studies also made use of model stacking, whereby the input of a model is the output of another trained model. This may impact the model's ability to generalize well to different data by increasing the chances of overfitting. This is because the first model in a stacking configuration has already derived a representation of the data informed by test labels. This bias is distinct from using traditional unsupervised dimensionality reduction techniques to derive an input for a subsequent predictive model. Additionally, deep learning systems can be opaque, making it difficult to understand the first deep learning model's data representation and consequently the properties of the input used for the final predictive model.

### 4.2 Repeat experiments

Most studies implemented repeat experiments via cross‐validation, which can account for performance estimation variation caused by weight initialization stochasticity and fold splitting. While studies varied in the amount of data available for training, and consequently the number of folds they considered during cross‐validation, evidence of repeat experiments greatly increases the reliability of reported performance metrics. As highlighted in (Hutson, 2018), reproducibility is not guaranteed even when code is provided, making repeat experiments particularly important. Twenty‐five of the 55 considered articles did not employ repeat experiments, which reduces confidence in their reported results. A number of studies using repeat experiments reported only point estimates, which do not fully describe the range of performance metrics, potentially leading the reader to underestimate the variation in performance. Code inaccessibility exacerbates this issue, leaving the reader unclear as to the procedure followed. We again found no significant difference between accuracy metrics reported across repeat experiment procedures (repeat experiment studies = 87.944 ± 10.43%, non‐repeat experiment studies = 91.58 ± 5.025%), although this does not minimize the importance of carrying out repeat experiments. We recommend that researchers continue to employ repeat experiments and report their results with means and standard deviations.

### 4.3 Code availability

Most studies did not provide code. As detailed in (Wen et al., 2020), the principles of fairness, accountability, and transparency are of paramount importance for deep learning modelling studies, and code inaccessibility is a significant obstacle to their realization. The construction of deep learning systems requires many algorithmic decisions which can influence performance, introduce bias, and impact reproducibility. Deep learning models optimize an objective function over a set of arguments, meaning that any decisions taken in preprocessing and model construction can affect the capabilities of the system as a whole, and propagate subjective choices throughout ostensibly objective models (Hooker, 2021). For instance, several studies have examined algorithmic biases against underrepresented and/or marginalised groups (Bagdasaryan et al., 2019; Buolamwini & Gebru, 2018; Diakopoulos, 2015). Aside from domain‐specific benefits to code sharing, the larger scientific community has recently shifted towards open science frameworks, with several high‐profile journals requiring methodological transparency (Eglen et al., 2017; Stodden, 2011; Nature editorial policies, 2021; Science editorial policies, 2021). Therefore, we believe that code availability and transparent methodological descriptions are important aspects of deep learning experiments in this domain, independent of potential clinical applications. Within a patient‐care context, we underscore the importance of constructing reproducible systems to increase trust, both from a clinician and patient perspective. We further encourage the exploration of minimal Jupyter/Google Colab notebooks—and other literate programming tools—to enhance understanding and reproducibility (Eglen et al., 2017; Google, 2018; Kluyver et al., 2016). This would also encourage accountability by allowing researchers to examine pipelines interactively and to identify any potential ‘blind spots‘that the model authors may have overlooked in their modelling decisions (Wen et al., 2020). Additionally, because model training is often computationally intensive, having access to models trained in similar domains could enable transfer learning approaches, accelerating scientific discovery in this domain. Therefore, we recommend that authors share model weights and code to facilitate reproducibility and increase the potential for clinical translation.

### 4.4 Saliency and interpretability

We found many studies did not interrogate their presented models to ensure that relevant information is being used to make predictive decisions. Where irrelevant information is included, clinical utility will be severely limited. For example, when investigating patterns of Alzheimer's disease neurodegeneration, it is important to verify that factors such as skull thickness are not significantly weighted by the model. Even in cases where known irrelevant information can be removed by preprocessing, visual maps can draw attention to global patterns that may highlight the biases of models. As previously stated, algorithmic biases in predictive settings are concerning, and saliency methods can help researchers identify sources of bias. Additionally, attempting to understand the image features driving model predictions can help to relate new models to previous findings. These methods may also be used to generate new hypotheses and discover novel biomarkers, for example by highlighting neuroanatomical regions discriminative for particular conditions which may suggest they have mechanistic relevance 17 studies investigated neuroanatomical features driving model predictions via interpretability methods, thus increasing the potential to highlight sources of bias in model training.

Interpretability methods, however, do have several considerations that may limit their utility, requiring careful consideration of how best to understand opaque models. Most existing methods deriving a saliency map return an ‘importance’ value per pixel, which has no direct link to human‐interpretable neuroanatomy. Usually, this represents the degree of change in the output relative to a small perturbation in the input pixel, collapsing a potentially non‐linear relationship to single values. While it provides an empirical assessment of captured patterns and is a useful visual aid, it offers little interpretative value compared to coefficients returned by classical statistical models. The deep learning field in general has been historically focused on prediction as opposed to inference, meaning that the mechanistic understanding of relationship dynamics is often secondary to test accuracy. This is challenging in the context of discovery and clinical settings. Furthermore, saliency methods have their own limitations arising from their algorithmic derivation of importance, which can affect interpretation (Adebayo et al., 2018). Similarly, while counterfactuals are promising, they are difficult to empirically measure and require significant computational overhead. Nonetheless, interpretability efforts allow researchers to visually evaluate model ‘attention’, which can serve to increase confidence and reduce bias overall, a topic of concern regarding the application of models to society at large (Diakopoulos, 2015; Hooker, 2021).

### 4.5 Accuracy metrics, sample sizes, and data sources

We found that, overall, studies reported impressive predictive accuracies in their primary modelling questions (89.36 ± 8.694%)underscoring the potential of deep learning models to aid clinical decision making This makes the careful consideration of the principles outlined here all the more important. Despite observing no significant differences in accuracies stratified by questionnaire categories, we highlight the importance of applying these principles from a qualitative standpoint. Studies with high accuracies that have applied repeat experiments and carefully considered data representation strategies can elicit more trust. This trust can be further enhanced by making code available so that results can be reproduced, with the additional benefit of allowing researchers to apply trained models to their own data. One significant barrier to full reproducibility in this context is data privacy concerns, which may limit the potential for release of a fully reproducible paper.

On average, sample sizes were large, although there was a high degree of variation (828 ± 691). While there was no significant relationship between sample size and accuracy, it appears that there is a weak negative correlation between the two variables, even in spite of large database crossover between studies, with 44 studies making use of ADNI (*R* ^2^ = 0.055, *p* = 0.09). This provides evidence that increased sample size may help to reduce bias in performance estimation.

Additionally, we note that every study detailed their preprocessing pipeline textually and explicitly stated their database source. This is an essential aspect of data transparency that should continue to be universally embraced by future authors. Our observation of 44 studies of a total pool of 55 making use of the same database speaks to the importance of the ADNI consortium, but may also indicate that this cohort of patients is overrepresented in the literature. This may be an issue when considering site effects in neuroimaging studies (Bayer et al., 2022). Future efforts to diversify data sources in this domain are dependent on establishing accessible and robust data consent frameworks that represent different data demographics.

### 4.6 Future perspectives and commentary

This systematic literature review highlights areas of focus across modelling practices, transparency, and interpretability in the context of maximizing the potential for clinical utility and reproducibility. These points underscore long‐standing differences between deep learning and classical statistics, whereby the former is usually concerned with predictive performance and the latter with making inferential statements. The predictive imperative has led to numerous advances in image processing, with several state‐of‐the‐art approaches developed to address tasks not suited to classical statistics (LeCun et al., 1995; Simonyan & Zisserman, 2015). Neural networks have clear advantages where inferential dynamics are not a concern, and the reported accuracies of the considered research provide further evidence of this.

However, as deep learning becomes more readily applied to medical imaging domains, with potential consequences for patients, dichotomies of prediction versus inference should be retired, even where models have clear discriminative potential. Researchers can maximize potential clinical utility and improve the quality of patient care by embracing the principles of reproducibility, transparency, and interpretability for predictive models. This will increase the confidence in such methods and accelerate the path to future clinical integration. We summarise our key recommendations in Table 3.

**TABLE 3:** Key recommendations arising from the results of this systematic literature review, their benefits, and the risks associated with non‐adherence.

Key recommendations	Benefits	Risk(s) mitigated
Make well‐annotated code freely available	Improve chances of reproducibility Readers can better understand workflow Encourage accountability and transparency	Limit reproducibility efforts Models remain opaque
Employ repeat experiments	Improve confidence in model estimation Mitigate random weight initialisation	Risk reporting overfitted results Performance estimation inflation Diminished confidence in system overall
Employ interpretability methods	Validate that model is using relevant information Potential biomarker discovery Improve confidence in system overall	Models remain opaque Diminished confidence in system overall Unsure what information is being used by models
Consider specifics of data representation	Lessen chance of information leakage Ensure optimal voting strategies Cognisant of strengths and weaknesses of different representations	Information leakage is more likely to occur May not consider multiple voting strategies May not consider optimal representation given experimental context

**TABLE 1:** Tabular presentation of the studies considered for this systematic literature review.

Authors and citation	Modelling practices			Transparency	Interpretability	Database(s)	Sample size
	Data representation	Repeat experiments	Accuracy (%)	Code availability	Saliency		
Zou et al. (2017)	3D	Yes, repeated on same data split	69.15	No	No	ADHD‐200	730
Gorji & Kaabouch (2019)	2D	No	94.54	No	No	ADNI	600
Spasov et al. (2018)	2D	No	99.0	No	No	ADNI	376
Li & Liu (2019)	3D	Yes, cross‐validation	91.0	No	Yes, saliency map	ADNI	807
Liu et al. (2020)	3D	Yes, cross‐validation	88.9	No	No	ADNI	449
Li et al. (2017)	3D	Yes, cross‐validation	88.31	No	No	ADNI	428
Folego et al. (2020)	3D	Yes, cross‐validation	52.3	Yes, github.com/qfolego/alzheimers	Yes, saliency map	ADNI, CADD, AIBL, MIRIAD, OASIS	3368
Marzban et al. (2020)	3D	No	93.5	No	No	ADNI	406
Hosseini‐Asl et al. (2018)	3D	Yes, cross‐validation	99.31	No	No	ADNI	210
Gunawardena et al. (2017)	2D	No	96.0	No	No	ADNI	504
Basaia et al. (2019)	3D	No	99.0	No	No	ADNI, In‐house	1638
Tufail et al. (2020)	2D	Yes, cross‐validation	94.64	No	No	OASIS	494
Hu et al. (2020)	3D	Yes, cross‐validation	70.68	No	No	NUSDAST, IMH	499
Cheng et al. (2017)	3D	No	87.15	No	No	ADNI	428
Nanni et al. (2020)	3D	No	NA	No	No	ADNI, S509	773
Lin et al. (2018)	2D	Yes, cross‐validation	79.9	No	No	ADNI	818
Billones et al. (2016)	2D	No	91.85	No	No	ADNI	NA
Barbaroux et al. (2020)	2D	Yes, cross‐validation	92.16	No	No	ADNI	589
Yigit & Işik (2020)	2D	No	83.0	No	No	OASIS, MIRIAD	485
Pan et al. (2020)	2D	Yes, cross‐validation	84.0	No	No	ADNI	787
Ahmed et al. (2020)	2D	No	90.73	No	No	GARD, ADNI	NA
Ortiz‐Su'arez et al. (2017)	2D	Yes, cross‐validation	85.0	No	Yes, feature map	OASIS	84
Aderghal et al. (2017)	2D	No	91.41	No	No	ADNI	815
Lian, Liu, Zhang, & Shen (2020)	3D	No	91.1	No	Yes, saliency map	ADNI	1458
Li, Li, Elahifasaee, & Liu (2021)	3D	Yes, cross‐validation	85.9	No	Yes, saliency map	ADNI	807
Cui & Liu (2018)	3D	Yes, cross‐validation	86.98	No	No	ADNI	231
Aderghal et al. (2020)	2D	No	91.86	No	No	ADNI	1551
Böhle et al. (2019)	3D	No	87.90	Yes, github.com/moboehle/Pytorch‐LRP	Yes, saliency map	ADNI	344
Sarraf et al. (2019)	2D	Yes, cross‐validation	99.9	Yes, github.com/samansarraf/MCADNNet	Yes, feature map	ADNI	1076
Liu, Cheng, Wang, et al. (2018)	3D	Yes, cross‐validation	93.26	No	Yes, saliency map	ADNI	397
Zhang et al. (2020)	3D	Yes, cross‐validation	85.0	No	Yes, feature map	In‐house	60
Lee & Ellahi (2019)	2D	Yes, cross‐validation	98.74	No	No	OASIS, ADNI	1235
Qiu et al. (2020)	3D	Yes, repeated on same data split	96.8	Yes, github.com/vkola‐lab/brain2020	Yes, feature map	ADNI, AIBL, PH, NACC	1483
Spasov et al. (2019)	3D	Yes, repeated on different data splits	100.0	Yes, github.com/simeon‐spasov/MCI	No	ADNI	785
Sun et al. (2020)	3D	Yes, cross‐validation	84.0	No	No	ADNI	132
Oh et al. (2020)	3D	Yes, cross‐validation	70.0	No	No	BGS, COBRE, MCICS, NMCH, NUSDAST	873
Lian, Liu, Pan, & Shen (2020)	3D	No	91.9	No	Yes, feature map	ADNI, AIBL	1977
Cui & Liu (2019b)	3D	Yes, cross‐validation	91.33	No	Yes, saliency map	ADNI	830
Mendoza‐Léon et al. (2020)	2D	No	89.16	No	No	OASIS	174
Pelka et al. (2020)	2D	Yes, cross‐validation	90.0	No	Yes, saliency map	Heinz Nixdorf Recall, ADNI	744
Li & Liu (2018)	3D	Yes, cross‐validation	89.5	No	Yes, feature map	ADNI	831
Bae et al. (2021)	3D	No	82.4	No	Yes, saliency map	ADNI	450
Cui & Liu (2019a)	3D	Yes, cross‐validation	92.29	No	No	ADNI	811
Liu, Zhang, Nie, et al. (2018)	3D	No	90.56	No	No	ADNI, MIRIAD	856
Liu, Zhang, Adeli, & Shen (2018)	3D	Yes, cross‐validation	92.75	No	No	ADNI, MIRIAD	1526
Al‐Khuzaie et al. (2021)	2D	No	99.3	No	No	OASIS	240
Zhang et al. (2021)	3D	No	97.35	No	No	ADNI	968
Hu et al. (2021)	3D	No	93.45	Yes, github.com/BigBug‐NJU/FTD AD transfer	Yes, feature map	ADNI, NIFD	1797
Herzog & Magoulas (2021)	2D	No	90.0	No	No	ADNI	750
Yee et al. (2021)	3D	Yes, cross‐validation	88.0	No	No	ADNI, AIBL, OASIS, MIRIAD	3577
Mukhtar & Farhan (2020)	2D	No	79.9	No	No	ADNI	818
Bae et al. (2020)	2D	Yes, cross‐validation	89.0	No	No	ADNI, SNUBH	390
Nigri et al. (2020)	2D	No	NA	No	Yes, saliency map	ADNI, AIBL	826
Li et al. (2021)	2D	Yes, cross‐validation	99.87	No	No	In‐house	212
Kiryu et al. (2019)	2D	No	96.8	No	No	In‐house	419

## 5 LIMITATIONS

This work reviewed studies from two database sources but is not guaranteed to have evaluated all available relevant research. This study also did not consider studies making use of functional neuroimaging data sources, which comprise a large corpus of research. We did not endeavour to comprehensively identify potential information leakage—an in‐depth consideration of this concept is explored in (Wen et al., 2020). Additionally, while we encourage the use of interpretability methods, we acknowledge the multiple drawbacks which may limit their utility and application. We further note that it is difficult to identify a unified set of optimal experimental parameters across every context—our commentary is designed to draw attention to the limitations arising from specific procedures and encourage researchers to carry out experiments that mitigate these issues as much as possible. We also note that fair comparison of reported accuracies across a myriad of diverse studies is extremely challenging.

Finally, we have endeavoured to ensure that our evaluation is neither reflective of overall study quality nor reductive with respect to the three nuanced principles introduced—our binary descriptors are intended to serve as a vehicle to discuss important concepts and to encourage continued careful research into brain conditions using CNN‐based predictive models.

## 6 CONCLUSION

We conducted a systematic literature review of 55 studies carrying out CNN‐based predictive modelling of brain disorders using structural brain imaging data and evaluated them in the context of their modelling practices, transparency, and interpretability. We provided recommendations that we believe will increase the potential clinical value of deep learning systems in this domain. Careful consideration of these concepts can help to inform a clinical framework that can effectively incorporate deep learning into diagnostic and prognostic systems, improving patient care.

## AUTHOR CONTRIBUTIONS

Shane O'Connell, Dara M. Cannon, and Pilib Ó Broin conceived the study; Shane O'Connell performed the literature search, analysed the data, and wrote the manuscript; Dara M. Cannon and Pilib Ó Broin provided feedback and revisions.

## CONFLICT OF INTEREST STATEMENT

All authors report no competing interests.

## Data Availability

All studies in this systematic literature review are accessible via PubMed and Web of Science.

## References

[hbm26521-bib-0001] Adebayo, J. , Gilmer, J. , Muelly, M. , Goodfellow, I. , Hardt, M. , & Kim, B. (2018). Sanity checks for saliency maps. *arXiv preprint arXiv:1810.03292* .

[hbm26521-bib-0002] Aderghal, K. , Afdel, K. , Benois‐Pineau, J. , & Gw'ena¨elle Catheline . (2020). Improving Alzheimer's stage categorization with convolutional neural network using transfer learning and different magnetic resonance imaging modalities. Heliyon, 6(12), e05652. 10.1016/j.heliyon.2020.e05652 33336093 PMC7733012

[hbm26521-bib-0003] Aderghal, K. , Benois‐Pineau, K. A. , & Catheline, G. (2017). FuseMe: Classification of sMRI images by fusion of deep CNNs in 2D+e projections. CBMI, 34, 1–7. 10.1145/3095713.3095749

[hbm26521-bib-0004] Ahmed, S. , Kim, B. C. , Lee, K. H. , Jung, H. Y. , & for the Alzheimer's Disease Neuroimaging Initiative . (2020). Ensemble of ROI‐based convolutional neural network classifiers for staging the Alzheimer disease spectrum from magnetic resonance imaging. PLoS One, 15(12), e0242712. 10.1371/journal.pone.0242712 33290403 PMC7723284

[hbm26521-bib-0005] Al‐Khuzaie, F. E. K. , Bayat, O. , & Duru, A. D. (2021). Diagnosis of Alzheimer disease using 2D MRI slices by convolutional neural network. Applied Bionics and Biomechanics, 2021, e6690539. 10.1155/2021/6690539 PMC7872776 33623535

[hbm26521-bib-0006] American Psychiatric Association . (2013). Diagnostic and statistical manual of mental disorders: DSM‐5 (5th ed.)., American Psychiatric Association.

[hbm26521-bib-0007] Bae, J. , Stocks, J. , Heywood, A. , Jung, Y. , Jenkins, L. , Hill, V. , Katsaggelos, A. , Popuri, K. , Rosen, H. , Beg, M. F. , Wang, L. , & Alzheimer's Disease Neuroimaging Initiative . (2021). Transfer learning for predicting conversion from mild cognitive impairment to dementia of Alzheimer's type based on a three‐dimensional convolutional neural network. Neurobiology of Aging, 99, 53–64. 10.1016/j.neurobiolaging.2020.12.005 33422894 PMC7902477

[hbm26521-bib-0008] Bae, J. B. , Lee, S. , Jung, W. , Park, S. , Kim, W. , Hyunwoo, O. , Han, J. W. , Kim, G. E. , Kim, J. S. , & Kim, J. H. (2020). Identification of alzheimer's disease using a convolutional neural network model based on t1‐weighted magnetic resonance imaging. Scientific Reports, 10(1), 1–10. 33335244 10.1038/s41598-020-79243-9 PMC7746752

[hbm26521-bib-0009] Bagdasaryan, E. , Poursaeed, O. , & Shmatikov, V. (2019). Differential privacy has disparate impact on model accuracy. Advances in Neural Information Processing Systems, 32, 15479–15488.

[hbm26521-bib-0010] Barbaroux, H. , Feng, X. , Yang, J. , Laine, A. F. , & Angelini, E. D. (2020). Encoding human cortex using spherical CNNs: A study on Alzheimer's disease classification. In 2020 IEEE 17th international symposium on biomedical imaging (ISBI) (pp. 1322–1325). 10.1109/ISBI45749.2020.9098353

[hbm26521-bib-0011] Basaia, S. , Agosta, F. , Wagner, L. , Canu, E. , Magnani, G. , Santangelo, R. , & Filippi, M. (2019). Automated classification of Alzheimer's disease and mild cognitive impairment using a single MRI and deep neural networks. NeuroImage: Clinical, 21, 101645. 10.1016/j.nicl.2018.101645 30584016 PMC6413333

[hbm26521-bib-0012] Bayer, J. M. M. , Thompson, P. M. , Ching, C. R. K. , Liu, M. , Chen, A. , Panzenhagen, A. C. , Jahanshad, N. , Marquand, A. , Schmaal, L. , & Samann, P. G. (2022). Site effects how‐to and when: An overview of retrospective techniques to accommodate site effects in multi‐site neuroimaging analyses. Frontiers in Neurology, 13, 923988. 36388214 10.3389/fneur.2022.923988 PMC9661923

[hbm26521-bib-0013] Billones, C. D. , Demetria, O. J. L. D. , Hostallero, D. E. D. , & Naval, P. C. (2016). Demnet: A convolutional neural network for the detection of alzheimer's disease and mild cognitive impairment. In In 2016 IEEE region 10 conference (TENCON) (pp. 3724–3727). IEEE.

[hbm26521-bib-0014] Böhle, M. , Eitel, F. , Weygandt, M. , & Ritter, K. (2019). Layer‐wise relevance propagation for explaining deep neural network decisions in MRI‐based Alzheimer's disease classification. Frontiers in Aging Neuroscience, 11, 194. 10.3389/fnagi.2019.00194 PMC6685087 31417397

[hbm26521-bib-0015] Botvinik‐Nezer, R. , Holzmeister, F. , Camerer, C. F. , Dreber, A. , Huber, J. , Johannesson, M. , Kirchler, M. , Iwanir, R. , Mumford, J. A. , Alison Adcock, R. (2020). Variability in the analysis of a single neuroimaging dataset by many teams. Nature, 582(7810), 84–88. 32483374 10.1038/s41586-020-2314-9 PMC7771346

[hbm26521-bib-0016] Buolamwini, J. , & Gebru, T. (2018). Gender shades: Intersectional accuracy disparities in commercial gender classification. In Conference on fairness, accountability and transparency (pp. 77–91). PMLR.

[hbm26521-bib-0017] Carroll, B. J. (2013). Biomarkers in dsm‐5: Lost in translation. Australian & New Zealand Journal of Psychiatry, 47(7), 676–678. 23814152 10.1177/0004867413491162

[hbm26521-bib-0018] Cheng, D. , Liu, M. , Jianliang, F. , & Wang, Y. (2017). Classification of MR brain images by combination of multi‐CNNs for AD diagnosis, 10420, 1042042. 10.1117/12.2281808

[hbm26521-bib-0019] Collins, G. S. , Reitsma, J. B. , Altman, D. G. , & Moons, K. G. M. (2015). Transparent reporting of a multivariable prediction model for individual prognosis or diagnosis (tripod) the tripod statement. Circulation, 131(2), 211–219. 25561516 10.1161/CIRCULATIONAHA.114.014508 PMC4297220

[hbm26521-bib-0020] Cui, R. , & Liu, M. (2018). Hippocampus analysis based on 3D CNN for Alzheimer's disease diagnosis. In Tenth international conference on digital image processing (ICDIP 2018) (Vol. 10806, 108065). International Society for Optics and Photonics. 10.1117/12.2503194

[hbm26521-bib-0021] Cui, R. , & Liu, M. (2019a). Hippocampus analysis by combination of 3‐D DenseNet and shapes for Alzheimer's disease diagnosis. IEEE Journal of Biomedical and Health Informatics, 23(5), 2099–2107. 10.1109/JBHI.2018.2882392 30475734

[hbm26521-bib-0022] Cui, R. , & Liu, M. (2019b). RNN‐based longitudinal analysis for diagnosis of Alzheimer's disease. Computerized Medical Imaging and Graphics, 73, 1–10. 10.1016/j.compmedimag.2019.01.005 30763637

[hbm26521-bib-0023] Diakopoulos, N. (2015). Algorithmic accountability: Journalistic investigation of computational power structures. Digital Journalism, 3(3), 398–415.

[hbm26521-bib-0024] Eglen, S. J. , Marwick, B. , Halchenko, Y. O. , Hanke, M. , Sufi, S. , Padraig Gleeson, R. , Silver, A. , Davison, A. P. , Lanyon, L. , Abrams, M. , Wachtler, T. , Willshaw, D. J. , Pouzat, C. , & Poline, J. B. (2017). Toward standard practices for sharing computer code and programs in neuroscience. Nature Neuroscience, 20(6), 770–773. 28542156 10.1038/nn.4550 PMC6386137

[hbm26521-bib-0025] Folego, G. , Weiler, M. , Casseb, R. F. , Pires, R. , & Rocha, A. (2020). Alzheimer's disease detection through whole‐brain 3D‐CNN MRI. Frontiers in Bioengineering and Biotechnology, 8, 534592. 10.3389/fbioe.2020.534592 PMC7661929 33195111

[hbm26521-bib-0026] Furiea, G. S. K. , & Gisele, S. (2009). Biomarkers in neurology. Frontiers of Neurology and Neuroscience, 25, 55–61. 19478497 10.1159/000209475

[hbm26521-bib-0027] Goldacre , B., Morton, C. E. , & DeVito, N. J . (2019). Why researchers should share their analytic code, BMJ, 367, l6365. 10.1136/bmj.l6365 31753846

[hbm26521-bib-0028] Google . Colaboratory: Frequently asked questions. 2018.

[hbm26521-bib-0029] Gorji, H. T. , & Kaabouch, N. (2019). A deep learning approach for diagnosis of mild cognitive impairment based on MRI images. Brain Sciences, 9(9), 217. 10.3390/brainsci9090217 31466398 PMC6770590

[hbm26521-bib-0030] Grover, V. P. B. , Tognarelli, J. M. , Crossey, M. M. E. , Jane Cox, I. , Taylor‐Robinson, S. D. , & McPhail, M. J. W. (2015). Magnetic resonance imaging: Principles and techniques: Lessons for clinicians. Journal of Clinical and Experimental Hepatology, 5(3), 246–255. 26628842 10.1016/j.jceh.2015.08.001 PMC4632105

[hbm26521-bib-0031] Gunawardena, K. A. N. N. P. , Rajapakse, R. N. , & Kodikara, N. D. (2017). Applying convolutional neural networks for pre‐detection of alzheimer's disease from structural MRI data. In 2017 24th international conference on mechatronics and machine vision in practice (M2VIP) (pp. 1–7). 10.1109/M2VIP.2017.8211486

[hbm26521-bib-0032] Haibe‐Kains, B. , Adam, G. A. , Hosny, A. , Khodakarami, F. , Waldron, L. , Wang, B. , McIntosh, C. , Goldenberg, A. , Kundaje, A. , & Greene, C. S. (2020). Transparency and reproducibility in artificial intelligence. Nature, 586(7829), E14–E16. 33057217 10.1038/s41586-020-2766-y PMC8144864

[hbm26521-bib-0033] Herzog, N. J. , & Magoulas, G. D. (2021). Brain asymmetry detection and machine learning classification for diagnosis of early dementia. Sensors (Basel, Switzerland), 21(3), 778. 10.3390/s21030778 33498908 PMC7865614

[hbm26521-bib-0034] Hibar, D. P. , Westlye, L. T. , van Erp, T. G. M. , Rasmussen, J. , Leonardo, C. D. , Faskowitz, J. , Haukvik, U. K. , Hartberg, C. B. , Doan, N. T. , & Agartz, I. (2016). Subcortical volumetric abnormalities in bipolar disorder. Molecular Psychiatry, 21(12), 1710–1716. 26857596 10.1038/mp.2015.227 PMC5116479

[hbm26521-bib-0035] Hooker, S. (2021). Moving beyond “algorithmic bias is a data problem”. Patterns, 2(4), 100241. 10.1016/j.patter.2021.100241 33982031 PMC8085589

[hbm26521-bib-0036] Hosseini‐Asl, E. , Ghazal, M. , Mahmoud, A. , Aslantas, A. , Shalaby, A. M. , Casanova, M. F. , Barnes, G. N. , Gimel'farb, G. , Keynton, R. , & ElBaz, A. (2018). Alzheimer's disease diagnostics by a 3D deeply supervised adaptable convolutional network. Frontiers in Bioscience (Landmark Edition), 23, 584–596. 28930562 10.2741/4606

[hbm26521-bib-0037] Hu, J. , Qing, Z. , Liu, R. , Zhang, X. , Lv, P. , Wang, M. , Yang, W. , He, K. , Yang, G. , & Zhang, B. (2021). Deep learning‐based classification and voxel based visualization of frontotemporal dementia and Alzheimer's disease. Frontiers in Neuroscience, 14. 10.3389/fnins.2020.626154 PMC7858673 33551735

[hbm26521-bib-0038] Hu, M. , Sim, K. , Zhou, J. H. , Jiang, X. , & Guan, C. (2020). Brain MRI‐based 3D convolutional neural networks for classification of schizophrenia and controls. Annual International Conference of the IEEE Engineering in Medicine and Biology Society. IEEE Engineering in Medicine and Biology Society. Annual International Conference, 2020, 1742–1745. 10.1109/EMBC44109.2020.9176610 33018334

[hbm26521-bib-0039] Hutson, M. (2018). Artificial intelligence faces reproducibility crisis. Science, 359, 725–726. 29449469 10.1126/science.359.6377.725

[hbm26521-bib-0040] Jack, C. R., Jr. , Bernstein, M. A. , Fox, N. C. , Thompson, P. , Alexander, G. , Harvey, D. , Borowski, B. , Britson, P. J. , Whitwell, J. L. , & Ward, C. (2008). The alzheimer's disease neuroimaging initiative (adni): Mri methods. Journal of Magnetic Resonance Imaging, 27(4), 685–691. 18302232 10.1002/jmri.21049 PMC2544629

[hbm26521-bib-0041] James, S. L. , Abate, D. , Abate, K. H. , Abay, S. M. , Abbafati, C. , Abbasi, N. , Abbastabar, H. , Abd‐Allah, F. , Abdela, J. , Abdelalim, A. , & Abdollahpour, I. (2018). Global, regional, and national incidence, prevalence, and years lived with disability for 354 diseases and injuries for 195 countries and territories, 1990–2017: A systematic analysis for the global burden of disease study 2017. The Lancet, 392(10159), 1789–1858. 10.1016/S0140-6736(18)32279-7 PMC6227754 30496104

[hbm26521-bib-0042] Jenkinson, M. (2012). Christian F Beckmann, Timothy EJ Behrens, mark W Woolrich, and Stephen M smith. Fsl. Neuroimage, 62(2), 782–790. 21979382 10.1016/j.neuroimage.2011.09.015

[hbm26521-bib-0043] Kamnitsas, K. , Ledig, C. , Newcombe, V. F. J. , Simpson, J. P. , Kane, A. D. , Menon, D. K. , Rueckert, D. , & Glocker, B. (2017). Efficient multi‐scale 3d cnn with fully connected crf for accurate brain lesion segmentation. Medical Image Analysis, 36, 61–78. 27865153 10.1016/j.media.2016.10.004

[hbm26521-bib-0044] Keane, M. T. , & Smyth, B. (2020). Good counterfactuals and where to find them: A case‐based technique for generating counterfactuals for explainable AI (XAI). CoRR, abs/2005.13997. https://arxiv.org/abs/2005.13997

[hbm26521-bib-0045] Kiryu, S. , Yasaka, K. , Akai, H. , Nakata, Y. , Sugomori, Y. , Hara, S. , Seo, M. , Abe, O. , & Ohtomo, K. (2019). Deep learning to differentiate parkinsonian disorders separately using single midsagittal mr imaging: A proof of concept study. European Radiology, 29(12), 6891–6899. 31264017 10.1007/s00330-019-06327-0

[hbm26521-bib-0046] Kluyver, T. , Ragan‐Kelley, B. , P'erez, F. , Granger, B. , Bussonnier, M. , Frederic, J. , Kelley, K. , Hamrick, J. , Grout, J. , Corlay, S. , Ivanov, P. , Avila, D'a. , Abdalla, S. , & Willing, C. (2016). Jupyter notebooks: A publishing format for reproducible computational workflows. In F. Loizides & B. Schmidt (Eds.), Positioning and power in academic publishing: Players, agents and agendas (pp. 87–90). IOS Press.

[hbm26521-bib-0047] Kupfer, D. J. , First, M. B. , & Regier, D. A. (2008). A research agenda for DSM V. American Psychiatric Publisher.

[hbm26521-bib-0048] LeCun, Y. A. , Bottou, L. , Orr, G. B. , & Muller, K.‐R. (2012). Efficient backprop. In Neural networks: Tricks of the trade (pp. 9–48). Springer.

[hbm26521-bib-0049] LeCun, Y. , & Bengio, Y. (1995). Convolutional networks for images, speech, and time series. The Handbook of Brain Theory and Neural Networks, 3361(10), 1995.

[hbm26521-bib-0050] Lee, B. , Ellahi, W. , & Choi, J. (2019). Using deep CNN with data permutation scheme for classification of Alzheimer's disease in structural magnetic resonance imaging (sMRI). IEICE Transactions on Information and Systems, E102.D, 1384–1395. 10.1587/TRANSINF.2018EDP7393

[hbm26521-bib-0051] Lepri, B. , Oliver, N. , Letouz'e, E. , Pentland, A. , & Vinck, P. (2018). Fair, transparent, and accountable algorithmic decision‐making processes. Philosophy & Technology, 31(4), 611–627.

[hbm26521-bib-0052] Li, A. , Li, F. , Elahifasaee, F. , & Liu, M. (2021). Lichi Zhang, and Alzheimer's disease neuroimaging Initiative. Hippocampal shape and asymmetry analysis by cascaded convolutional neural networks for Alzheimer's disease diagnosis. Brain Imaging and Behavior, 15, 2330–2339. 10.1007/s11682-020-00427-y 33398778

[hbm26521-bib-0053] Li, F. , Cheng, D. , & Liu, M. (2017). Alzheimer's disease classification based on combination of multi‐model convolutional networks. IEEE International Conference on Imaging Systems and Techniques (IST). 10.1109/IST.2017.8261566

[hbm26521-bib-0054] Li, F. , & Liu, M. (2018). Alzheimer's disease diagnosis based on multiple cluster dense convolutional networks. Computerized Medical Imaging and Graphics, 70, 101–110. 10.1016/j.compmedimag.2018.09.009 30340094

[hbm26521-bib-0055] Li, F. , & Liu, M. (2019). A hybrid convolutional and recurrent neural network for hippocampus analysis in Alzheimer's disease. Journal of Neuroscience Methods, 323, 108–118. 10.1016/j.jneumeth.2019.05.006 31132373

[hbm26521-bib-0056] Li, Z. , Li, W. , Wei, Y. , Gui, G. , Zhang, R. , Liu, H. , Chen, Y. , & Jiang, Y. (2021). Deep learning based automatic diagnosis of first‐episode psychosis, bipolar disorder and healthy controls. Computerized Medical Imaging and Graphics, 89, 101882. 33684730 10.1016/j.compmedimag.2021.101882

[hbm26521-bib-0057] Lian, C. , Liu, M. , Pan, Y. , & Shen, D. (2020). Attention‐guided hybrid network for dementia diagnosis with structural MR images. IEEE Transactions on Cybernetics, 52, 1–12. 10.1109/TCYB.2020.3005859 PMC7855081 32721906

[hbm26521-bib-0058] Lian, C. , Liu, M. , Zhang, J. , & Shen, D. (2020). Hierarchical fully convolutional network for joint atrophy localization and Alzheimer's disease diagnosis using structural MRI. IEEE Transactions on Pattern Analysis and Machine Intelligence, 42(4), 880–893. 10.1109/TPAMI.2018.2889096 30582529 PMC6588512

[hbm26521-bib-0059] Lin, W. , Tong, T. , Gao, Q. , Guo, D. , Xiaofeng, D. , Yang, Y. , Guo, G. , Xiao, M. , Min, D. , Xiaobo, Q. , & The Alzheimer's Disease Neuroimaging Initiative . (2018). Convolutional neural networks‐based MRI image analysis for the Alzheimer's disease prediction from mild cognitive impairment. Frontiers in Neuroscience, 12, 777. 10.3389/fnins.2018.00777 PMC6231297 30455622

[hbm26521-bib-0060] Littlejohns, T. J. , Holliday, J. , Gibson, L. M. , Garratt, S. , Oesingmann, N. , AlfaroAlmagro, F. , Bell, J. D. , Boultwood, C. , Collins, R. , Conroy, M. C. , Crabtree, N. , Doherty, N. , Frangi, A. F. , Harvey, N. C. , Leeson, P. , Miller, K. L. , Neubauer, S. , Petersen, S. E. , & Sellors, J. (2020). The UK biobank imaging enhancement of 100,000 participants: Rationale, data collection, management and future directions. Nature Communications, 11(1), 1–12. 10.1038/s41467-020-15948-9 PMC7250878 32457287

[hbm26521-bib-0061] Liu, M. , Cheng, D. , Wang, K. , Wang, Y. , & Initiative, A's. D. N. (2018). Multi‐modality cascaded convolutional neural networks for Alzheimer's disease diagnosis. Neuroinformatics, 16(3–4), 295–308. 10.1007/s12021018-9370-4 29572601

[hbm26521-bib-0062] Liu, M. , Li, F. , Yan, H. , Wang, K. , Ma, Y. , Shen, L. , & Mingqing, X. (2020). A multi‐model deep convolutional neural network for automatic hippocampus segmentation and classification in Alzheimer's disease. NeuroImage, 208, 116459. 10.1016/j.neuroimage.2019.116459 31837471

[hbm26521-bib-0063] Liu, M. , Zhang, J. , Adeli, E. , & Shen, D. (2018). Landmark‐based deep multi‐instance learning for brain disease diagnosis. Medical Image Analysis, 43, 157–168. 10.1016/j.media.2017.10.005 29107865 PMC6203325

[hbm26521-bib-0064] Liu, M. , Zhang, J. , Nie, D. , Yap, P.‐T. , & Shen, D. (2018). Anatomical landmark based deep feature representation for MR images in brain disease diagnosis. IEEE Journal of Biomedical and Health Informatics, 22(5), 1476–1485. 10.1109/JBHI.2018.2791863 29994175 PMC6238951

[hbm26521-bib-0065] Markowetz, F. (2015). Five selfish reasons to work reproducibly. Genome Biology, 16(1), 1–4. 26646147 10.1186/s13059-015-0850-7 PMC4673789

[hbm26521-bib-0066] Marzban, E. N. , Eldeib, A. M. , Yassine, I. A. , Kadah, Y. M. , & for the Alzheimer's Disease Neurodegenerative Initiative . (2020). Alzheimer's disease diagnosis from diffusion tensor images using convolutional neural networks. PLoS One, 15(3), e0230409. 32208428 10.1371/journal.pone.0230409 PMC7092978

[hbm26521-bib-0067] Mendoza‐Léon, R. , Puentes, J. , Uriza, L. F. , & Marcela Hern'andez Hoyos . (2020). Singleslice Alzheimer's disease classification and disease regional analysis with supervised switching autoencoders. Computers in Biology and Medicine, 116, 103527. 10.1016/j.compbiomed.2019.103527 31765915

[hbm26521-bib-0068] Milham, M. P. , Cameron Craddock, R. , & Klein, A. (2017). Clinically useful brain imaging for neuropsychiatry: How can we get there? Depression and Anxiety, 34(7), 578–587. 28426908 10.1002/da.22627

[hbm26521-bib-0069] Mukhtar, G. , & Farhan, S. (2020). Convolutional neural network based prediction of conversion from mild cognitive impairment to alzheimer's disease: A technique using hippocampus extracted from mri. Advances in Electrical and Computer Engineering, 20(2), 113–122.

[hbm26521-bib-0070] Nanni, L. , Interlenghi, M. , Brahnam, S. , Salvatore, C. , Papa, S. , Nemni, R. , Castiglioni, I. , & The Alzheimer's Disease Neuroimaging Initiative . (2020). Comparison of transfer learning and conventional machine learning applied to structural brain MRI for the early diagnosis and prognosis of Alzheimer's disease. Frontiers in Neurology, 11, 576194. 10.3389/fneur.2020.576194 PMC7674838 33250847

[hbm26521-bib-0071] Nature editorial policies . (2021). https://www.nature.com/nature-portfolio/editorial-policies/reportingstandards.

[hbm26521-bib-0072] Nigri, E. , Ziviani, N. , Cappabianco, F. , Antunes, A. , & Veloso, A. (2020). Explainable deep cnns for mri‐based diagnosis of alzheimer's disease. In In 2020 international joint conference on neural networks (IJCNN) (pp. 1–8). IEEE.

[hbm26521-bib-0073] Oh, J. , Baek‐Lok, O. , Lee, K.‐U. , Chae, J.‐H. , & Yun, K. (2020). Identifying schizophrenia using structural MRI with a deep learning algorithm. Frontiers in Psychiatry, 11, 16. 10.3389/fpsyt.2020.00016 32116837 PMC7008229

[hbm26521-bib-0074] Ortiz‐Su'arez, J. M. , Ramos‐Poll'an, R'l. , & Romero, E. (2017). Exploring Alzheimer's anatomical patterns through convolutional networks. 12th international symposium on medical information processing and analysis, 10160, 101600Z. International Society for Optics and Photonics.

[hbm26521-bib-0075] Page, M. J. , McKenzie, J. E. , Bossuyt, P. M. , Boutron, I. , Hoffmann, T. C. , Mulrow, C. D. , Shamseer, L. , Tetzlaff, J. M. , Akl, E. A. , & Brennan, S. E. (2021). The prisma 2020 statement: An updated guideline for reporting systematic reviews. BMJ, 372, 71. 10.1136/bmj.n71 PMC8005924 33782057

[hbm26521-bib-0076] Pan, D. , Zeng, A. , Jia, L. , Huang, Y. , Frizzell, T. , & Song, X. (2020). Early detection of Alzheimer's disease using magnetic resonance imaging: A novel approach combining convolutional neural networks and ensemble learning. Frontiers in Neuroscience, 14, 259. 10.3389/fnins.2020.00259 32477040 PMC7238823

[hbm26521-bib-0077] Pelka, O. , Friedrich, C. M. , Nensa, F. , Monninghoff, C. , Bloch, L. , Jockel, K. H. , Schramm, S. , Hoffmann, S. S. , Winkler, A. , Weimar, C. , Jokisch, M. , & Alzheimer's Disease Neuroimaging Initiative . (2020). Sociodemographic data and APOE‐4 augmentation for MRI‐based detection of amnestic mild cognitive impairment using deep learning systems. PLoS One, 15(9), e0236868. 10.1371/journal.pone.0236868 32976486 PMC7518632

[hbm26521-bib-0078] Qiu, S. , Joshi, P. S. , Miller, M. I. , Xue, C. , Zhou, X. , Karjadi, C. , Chang, G. H. , Joshi, A. S. , Dwyer, B. , Zhu, S. , Kaku, M. , Zhou, Y. , Alderazi, Y. J. , Swaminathan, A. , Kedar, S. , Saint‐Hilaire, M.‐H. , Auerbach, S. H. , Yuan, J. , Sartor, E. A. , … Kolachalama, V. B. (2020). Development and validation of an interpretable deep learning framework for Alzheimer's disease classification. Brain: A Journal of Neurology, 143(6), 1920–1933. 10.1093/brain/awaa137 32357201 PMC7296847

[hbm26521-bib-0079] Reuter, M. , Schmansky, N. J. , Rosas, H. D. , & Fischl, B. (2012). Within‐subject template estimation for unbiased longitudinal image analysis. NeuroImage, 61(4), 1402–1418. 10.1016/j.neuroimage.2012.02.084 22430496 PMC3389460

[hbm26521-bib-0080] Roh, J. H. , Qiu, A. , Seo, S. W. , Soon, H. W. , Kim, J. H. , Kim, G. H. , Kim, M.‐J. , Lee, J.‐M. , & Na, D. L. (2011). Volume reduction in subcortical regions according to severity of alzheimer's disease. Journal of Neurology, 258(6), 1013–1020. 21240517 10.1007/s00415-010-5872-1

[hbm26521-bib-0081] Sarraf, S. , Desouza, D. D. , Anderson, J. , Saverino, C. , & Alzheimer's Disease Neuroimaging Initiative . (2019). MCADNNet: Recognizing stages of cognitive impairment through efficient convolutional fMRI and MRI neural network topology models. IEEE Access: Practical Innovations, Open Solutions, 7, 155584–155600. 10.1109/ACCESS.2019.2949577 32021737 PMC6999050

[hbm26521-bib-0082] Science editorial policies . (2021). https://www.science.org/content/page/science-journals-editorial-policies.

[hbm26521-bib-0083] Selvaraju, R. R. , Das, A. , Vedantam, R. , Cogswell, M. , Parikh, D. , & Batra, D. (2016). Grad‐cam: Why did you say that? arXiv Preprint. arXiv:1611.07450.

[hbm26521-bib-0084] Shah, J. , & Scott, J. (2016). Concepts and misconceptions regarding clinical staging models. Journal of Psychiatry & Neuroscience, 41(6), E83–E84. 10.1503/jpn.160196 PMC5082515 27768563

[hbm26521-bib-0085] Karen Simonyan and Andrew Zisserman . Very deep convolutional networks for large‐scale image recognition, 2015.

[hbm26521-bib-0086] Spasov, S. E. , Passamonti, L. , Duggento, A. , Lio, P. , & Toschi, N. (2018). A multimodal convolutional neural network framework for the prediction of Alzheimer's disease. *Annual international conference of the IEEE engineering in medicine and biology society* . IEEE Engineering in Medicine and Biology Society. Annual International Conference, 2018, 1271–1274. 10.1109/EMBC.2018.8512468 30440622

[hbm26521-bib-0087] Spasov, S. , Passamonti, L. , Duggento, A. , Pietro Li'o , & Toschi, N. (2019). A parameter‐efficient deep learning approach to predict conversion from mild cognitive impairment to Alzheimer's disease. NeuroImage, 189, 276–287. 10.1016/j.neuroimage.2019.01.031 30654174

[hbm26521-bib-0088] Victoria C Stodden . Trust your science? Open your data and code. 2011. AMStat News.

[hbm26521-bib-0089] Sun, J. , Yan, S. , Song, C. , & Han, B. (2020. ISSN 1861‐6429). Dual‐functional neural network for bilateral hippocampi segmentation and diagnosis of Alzheimer's disease. International Journal of Computer Assisted Radiology and Surgery, 15(3), 445–455. 10.1007/s11548-019-02106-w 31883064

[hbm26521-bib-0090] Taber, K. H. , Hurley, R. A. , & Yudofsky, S. C. (2010). Diagnosis and treatment of neuropsychiatric disorders. Annual Review of Medicine, 61, 121–133. 10.1146/annurev.med.051408.105018 19824816

[hbm26521-bib-0091] Tufail, A. B. , Zhang, Q.‐N. , & Ma, Y.‐K. (2020). Binary classification of Alzheimer disease using sMRI imaging modality and deep learning. Journal of Digital Imaging, 33(5), 1073–1090. 10.1007/s10278-019-00265-5 32728983 PMC7573078

[hbm26521-bib-0092] Ueda, M. , Ito, K. , Kai, W. , Sato, K. , Taki, Y. , Fukuda, H. , & Aoki, T. (2019). An age estimation method using 3d‐cnn from brain mri images. In 2019 IEEE 16th international symposium on biomedical imaging (ISBI 2019) (pp. 380–383). IEEE.

[hbm26521-bib-0093] Walsh, I. , Fishman, D. , Garcia‐Gasulla, D. , Titma, T. , Pollastri, G. , Harrow, J. , Psomopoulos, F. E. , & Tosatto, S. C. E. (2021). Dome: Recommendations for supervised machine learning validation in biology. Nature Methods, 18(10), 1122–1127. 34316068 10.1038/s41592-021-01205-4

[hbm26521-bib-0094] Weiss, K. , Khoshgoftaar, T. M. , & Wang, D. D. (2016). A survey of transfer learning. Journal of Big Data, 3(1), 1–40.

[hbm26521-bib-0095] Wen, J. , Thibeau‐Sutre, E. , Diaz‐Melo, M. , Samper‐Gonz'alez, J. , Routier, A. , Bottani, S. , Dormont, D. , Durrleman, S. , Burgos, N. , & Colliot, O. (2020). Convolutional neural networks for classification of alzheimer's disease: Overview and reproducible evaluation. Medical Image Analysis, 63, 101694. 32417716 10.1016/j.media.2020.101694

[hbm26521-bib-0096] Yam, J. Y. F. , & Chow, T. W. S. (2000). A weight initialization method for improving training speed in feedforward neural network. Neurocomputing, 30(1–4), 219–232.

[hbm26521-bib-0097] Yee, E. , Ma, D. , Popuri, K. , Wang, L. , Beg, M. F. , & for the Alzheimer's Disease Neuroimaging Initiative, and and the Australian Imaging Biomarkers and Lifestyle flagship study of ageing . (2021). Construction of MRI‐based Alzheimer's disease score based on efficient 3D convolutional neural network: Comprehensive validation on 7,902 images from a multi‐center dataset. Journal of Alzheimer's Disease: JAD, 79(1), 47–58. 10.3233/JAD200830 33252079 PMC9159475

[hbm26521-bib-0098] Yigit, A. , & Işik, Z. (2020). Applying deep learning models to structural MRI for stage prediction of Alzheimer's disease. Turkish Journal of Electrical Engineering and Computer Sciences, 28, 196–210. 10.3906/elk-1904-172

[hbm26521-bib-0099] Zhang, J. , Li, X. , Li, Y. , Wang, M. , Huang, B. , Yao, S. , & Shen, L. (2020). Three dimensional convolutional neural network‐based classification of conduct disorder with structural MRI. Brain Imaging and Behavior, 14(6), 2333–2340. 10.1007/s11682-019-00186-5 31538277

[hbm26521-bib-0100] Zhang, J. , Zheng, B. , Gao, A. , Feng, X. , Dong, L. , & Long, X. (2021). A 3D densely connected convolution neural network with connection‐wise attention mechanism for Alzheimer's disease classification. Magnetic Resonance Imaging, 78, 119–126. 10.1016/j.mri.2021.02.001 33588019

[hbm26521-bib-0101] Zhang, Z. , Beck, M. W. , Winkler, D. A. , Huang, B. , Sibanda, W. , & Goyal, H. (2018). Opening the black box of neural networks: Methods for interpreting neural network models in clinical applications. Annals of Translational Medicine, 6(11), 216. 30023379 10.21037/atm.2018.05.32 PMC6035992

[hbm26521-bib-0102] Zou, L. , Zheng, J. , Miao, C. , Mckeown, M. J. , & Wang, Z. J. (2017). 3D CNN based automatic diagnosis of attention deficit hyperactivity disorder using functional and structural MRI. IEEE Access, 5, 23626–23636. 10.1109/ACCESS.2017.2762703

